# Formation and characteristics of mixed lipid/ polymer membranes on a crystalline surface-layer protein lattice

**DOI:** 10.1116/1.5132390

**Published:** 2020-01-16

**Authors:** Christian Czernohlavek, Bernhard Schuster

**Affiliations:** Department of NanoBiotechnology, Institute for Synthetic Bioarchitectures, University of Natural Resources and Life Sciences, Vienna, Muthgasse 11, 1190 Vienna, Austria

## Abstract

The implementation of self-assembled biomolecules on solid materials, in particular, sensor and electrode surfaces, gains increasing importance for the design of stable functional platforms, bioinspired materials, and biosensors. The present study reports on the formation of a planar hybrid lipid/polymer membrane on a crystalline surface layer protein (SLP) lattice. The latter acts as a connecting layer linking the biomolecules to the inorganic base plate. In this approach, chemically bound lipids provided hydrophobic anchoring moieties for the hybrid lipid/polymer membrane on the recrystallized SLP lattice. The rapid solvent exchange technique was the method of choice to generate the planar hybrid lipid/polymer membrane on the SLP lattice. The formation process and completeness of the latter were investigated by quartz crystal microbalance with dissipation monitoring and by an enzymatic assay using the protease subtilisin A, respectively. The present data provide evidence for the formation of a hybrid lipid/polymer membrane on an S-layer lattice with a diblock copolymer content of 30%. The hybrid lipid/polymer showed a higher stiffness compared to the pure lipid bilayer. Most interestingly, both the pure and hybrid membrane prevented the proteolytic degradation of the underlying S-layer protein by the action of subtilisin A. Hence, these results provide evidence for the formation of defect-free membranes anchored to the S-layer lattice.

## I Introduction

Bioinspired systems attract much attention nowadays because of their building blocks with unique, predictable, and tunable properties and diversity. Moreover, the feasibility to fabricate ultrathin two-dimensional structures in the square-micrometer range with an astonishing variety of functionalities at the nanoscale is of utmost importance for designing novel devices.^1–5^ Furthermore, by nature, these natural building blocks, e.g., lipids and proteins, spontaneously self-assemble into supramolecular structures.^6,7^ One of the most important templates for this bottom-up approach is the cell envelope structure of archaea.^8,9^ This is a complex layered structure and comprises a cell membrane, which separates the interior of all cells from the outside environment.^4,10,11^ The membrane consists of amphiphilic lipid molecules, which outnumber all other involved biomolecules. In addition, a monomolecular array of protein subunits forms the outermost surface layer (S-layer) at almost all archaea and many bacteria.^4,12^ The importance of cell membranes in biological systems as a barrier structure, as preserver of the physical integrity of the cell, and as host for integral membrane proteins has prompted the development of model membrane platforms that recapitulate fundamental aspects of membrane biology.^13^ The lipid bilayer environment is of utmost importance for integral membrane proteins such as (ion) channels, proton pumps, and (G protein-coupled) receptors, which are responsible for carrying out specific membrane functions.^14–16^

Supported lipid mono- and bilayers represent one of the most promising classes of model membranes.^5,6,17–20^ The immobilization of a planar lipid membrane on a solid support enables its characterization by a wide range of surface-sensitive analytical tech-niques.^6,20,21^ Moreover, supported lipid membranes are increasingly able to mimic fundamental properties of natural cell membranes, including fluidity, electrical sealing, and hosting integral membrane proteins.^22,23^ Most importantly, the S-layer protein (SLP) lattice as a tethering and anchoring structure in-between the solid support and the lipid membrane significantly improves the integrity of the latter and its functioning as reconstitution matrix for integral membrane proteins. ^6,24–27^

However, the challenge of integrating nonliving systems with biological ones led to problems associated with material interfacing and compatibility, as well as biological issues, such as viability and stability.^5,28^ Moreover, water is necessary to maintain biological units but has adverse effects on engineering and electronic components. Although newly developed methods improved the durability of pure lipid membranes, it is still a big challenge to significantly extend their shelf life.^6,21,29,30^ An innovative approach in this context is the generation of spherical mixed hybrid lipid/polymer membranes, which have so far carried out by the combined selfassembly of phospholipids and amphiphilic block copolymers. These hybrid vesicles revealed improved and modulated membrane bulk properties, e.g., permeability, toughness, elevated stability against mechanical stress, and air exposure.^31–34^ Based on these observations, the strategy to blend phospholipids with block copolymers for the generation of planar hybrid devices is anticipated to be highly promising, on the one hand, to extend the longevity and, on the other hand, to preserve the fluidity and thus functionality of planar bioinspired architectures.

So far, planar solid-supported membranes comprising block copolymers have been generated either by vesicle spreading^35,36^ or by the successive transfer of two polymeric monolayers by the Langmuir-Blodgett and Langmuir-Schaefer techniques.^37–39^ Bilayer membranes comprising binary lipid/block copolymer mixtures have been generated by the substrate-mediated fusion of small hybrid lipid/polymer vesicles on silica surfaces.^34,36^ Studying this self-consistent set of bilayer/monolayer morphologies on micropatterned surfaces revealed that in all cases, despite the large thickness mismatch in the hydrophobic core of polymer and lipid, the hybrid membranes do not exhibit microscopic phase separation. Moreover, fluorescence recovery after photobleaching (FRAP) measurements confirmed the onset of lateral fluidity at a fractional lipid content of approximately 70%, beyond which the probe diffusion constant climbs rapidly, increasing by 2 orders of magnitude.^34^

**Fig. 1:**
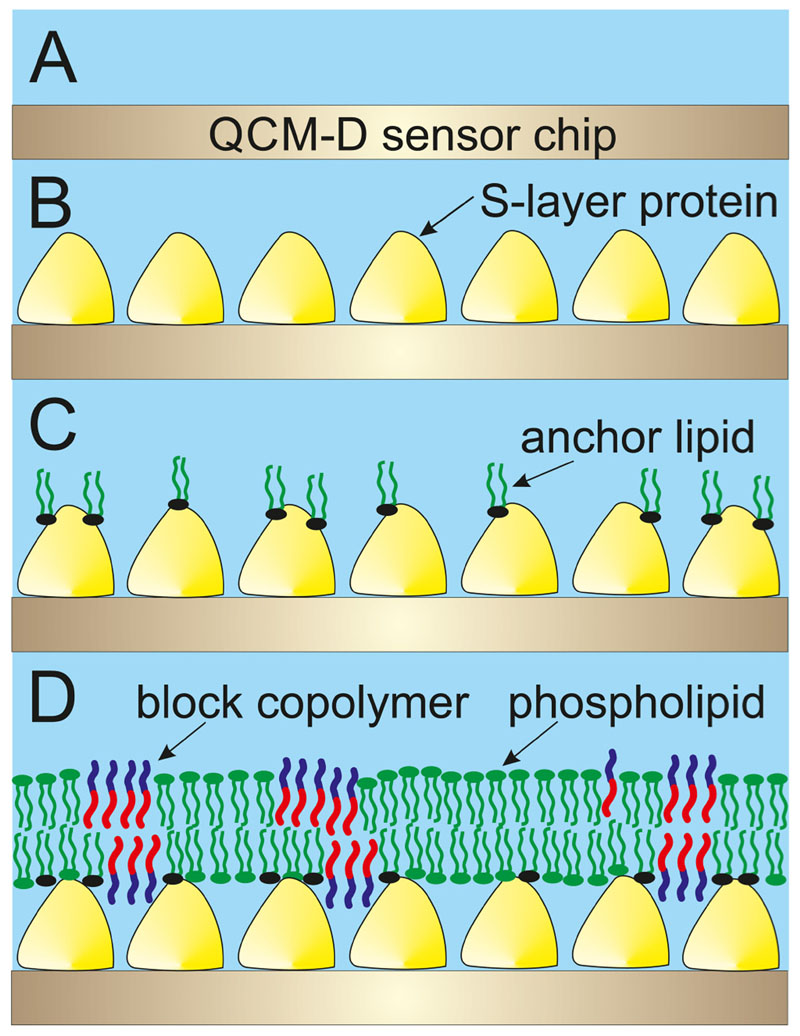
Schematic drawing of the formation of the mixed lipid/block copolymer layer on an S-layer lattice. (a) Gold-coated QCM-D sensor crystal onto which (b) S-layer protein was recrystallized. (c) Chemically coupled phospholipids on the S-layer protein provided hydrophobic anchoring points. The structure shown in (c) was incubated with an ethanolic solution comprising lipid and polymer. (d) Finally, this solution was rapidly exchanged by buffer to form a mixed lipid/polymer membrane on the S-layer lattice.

The present study is the first attempt to investigate the generation of planar hybrid lipid/block copolymer membranes with a significant polymer percentage on an SLP lattice by the rapid solvent exchange (RSE) technique (Fig. 1). The SLP recrystallization and the formation of the anchored planar hybrid lipid/polymer layer on the top of it were investigated by the surface-sensitive acoustical method quartz crystal microbalance with dissipation monitoring (QCM-D). Finally, the S-layer supported hybrid lipid/polymer membrane was incubated with the protease subtilisin A. This enzymatic assay allowed to determine by QCM-D whether the blended membrane is closed and hole-free and hence protects the underlying SLP from enzymatic degradation by subtilisin A or not.

## II Experiment

Growth, cell wall preparation, and extraction of the SLP SbpA isolated from *Lysinibacillus sphaericus* CCM 2177 (Czech Collection of Microorganisms) were performed as previously described.^40^ SbpA stock solution was diluted with 0.5 mM Tris (*p*H = 9) containing 10 mM CaCl_2_ to a final concentration of 0.1 mg/ml (buffer A).

Phosphate-buffered saline (PBS) buffer for the anchoring procedure was prepared by mixing monobasic and dibasic sodium dihydrogen phosphate solutions in distinct ratios and brought to the desired *p*H by titration with HCl (Merck). 400 mM 1-ethyl-3-(3-dimethylaminopropyl) carbodiimide (EDC) and 200 mM sulfo-*N*-hydroxy-sulfo-succinimide sodium salt (S-NHS) (both from Sigma-Aldrich) were stored as aliquots in 50 mM PBS buffer at *p*H 6.5 at -20 °C.

Poly(butadiene-b-ethylene oxide) (PBD-PEO; rich in 1,2 microstructure; M_w_= 1050g/mol; polydispersity index of 1.09) with the average molecular weights of 650 and 400 g/mol for the PBD_12_ and PEO_9_ blocks, respectively, was purchased from Polymer Source (Dorval, Quebec, Canada; No. P10054A-BdEO). All lipids were purchased from Avanti Polar Lipids (Alabaster, AL, USA). For the anchoring procedure, 1,2-dimyristoyl-sn-glycero-3-phosphoetha-nolamine (DMPE; M_w_ = 636 g/mol) in 50 mM PBS buffer at *p*H 7.4 containing 0.003% Triton X-100 (Fluka) was used. For the RSE technique, 1,2-dioleoyl-sn-glycero-3-phosphocholine (DOPC; M_w_ = 786 g/mol) or a mixture of 30:70 mol. % PBD-PEO:DOPC was solved in 70% ethanol. The flushing buffer for RSE was 10 mM 4-(2-hydroxyethyl)-1-piperazineethanesulfonic acid (HEPES) at *p*H 9.0.^41^

The generation of the model lipid membrane occurred by the RSE technique. Briefly, the S-layer lattice with the chemically bound DMPE molecules [Fig. 1(c)] was first flushed with 70% ethanol. Subsequently, a 10 mM solution of DOPC:PBD-PEO (70:30 mol. %) in 70% ethanol was allowed to interact with the modified S-layer lattice for 10 min at 21 °C.^41^ Higher content of ethanol was not used due to structural loss of SbpA.^42^ Afterward, the ethanol lipid/polymer mixture was rapidly displaced by a large amount of HEPES buffer. For RSE, one has to keep in mind that the change from an aqueous to an organic phase and *vice versa* is known to show a dramatic shift in frequency and dissipation due to the so-called solvent effect.^43^

QCM-D measurements were carried out with a Q-Sense E4 device (electronic unit) equipped with four standard flow modules (QSense, Biolin Scientific). The QSX 301 gold-covered crystal sensors have a fundamental frequency of 4.95 MHz (Q-Sense). After cleaning, the sensors were immediately mounted in the flow modules of the device. The flow rate was 100 *μ*l/min. The shift in frequency (Δf) and dissipation (ΔD) was recorded using the Q-SOFT 401 software. The presented results correspond to the fifth overtone at a temperature of 21.00 ± 0.02 °C. If not other indicated, all given data are mean values of two independent measurements.

For the subtilisin A protease assay, the protease subtilisin A from *Bacillus licheniformis* (M_w_ = 27 kDa; Sigma-Aldrich; P8038) was dissolved in buffer A at a concentration of 0.1 mg/ml. Subtilisin A, a member of the serine S8 endoproteinase family^44^ has broad specificity, hydrolyzes native and denatured proteins, and is active under alkaline conditions. To prove the formation of a hole-free membrane comprised of phospholipids with and without diblock copolymer on the underlying SLP lattice, the subtilisin A solution was passed over the hybrid lipid/polymer layer. In general, when Δ*f* becomes more negative, the biomolecules attach the surface of the QCM-D sensor. In turn, a detachment of biomolecules (in this case, hydrolyzed SLP) can be concluded whenever Δ*f* becomes more positive.

## III Results And Discussion

### A Plain S-layer lattice

**Fig. 2:**
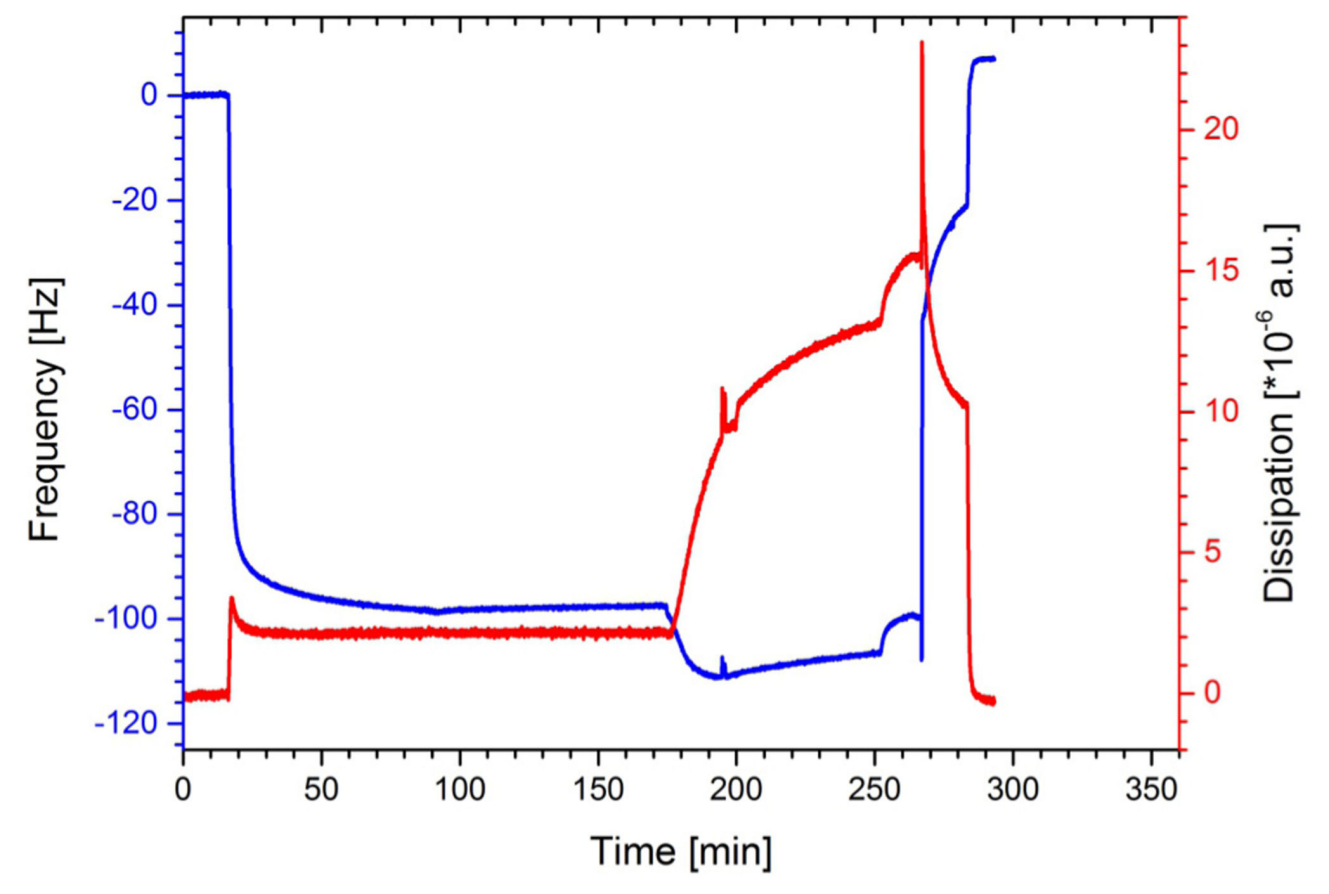
Shift in frequency (blue) and dissipation (red) during the formation process of the S-layer (SbpA) lattice and the subsequent treatment with subtilisin A (at time = 176 min).

After the formation of a stable baseline, SbpA was injected at the time point 16 min onto the gold surface of the QCM-D sensor. The frequency immediately dropped down and reached after a few minutes almost the maximum in —Δf (Fig. 2). The average for the final shift in frequency was calculated to be —90.8 ± 4.6 Hz (n = 9). The recrystallization of SbpA to form a coherent lattice on the gold surface [schematically depicted in Fig. 1(b)] took between 60 and 90 min. The ΔD signal showed immediately after the adsorption of SbpA a strong increase to a maximum, and finally the AD value levelled off to 2.0 ± 0.4 x 10^—6^ a.u. (n = 3). Both parameters determined by QCM-D provide evidence for a closed and rigid S-layer lattice on the gold surface of the quartz sensor crystal. The shape of the curves for both frequency and dissipation (Fig. 2) is well known from the literature to correspond to the formation of a crystalline monomolecular SbpA lattice.^41,45–47^

As a reference experiment and, in particular, to determine the enzymatic impact of subtilisin A on the crystalline SbpA lattice, the protease subtilisin A was added at the time point 176 min to the preformed S-layer lattice. In the following, Δf decreased further, which can be attributed to the interaction and/or adsorption of subtilisin A on the S-layer lattice. At the same time, Δ*D* increased dramatically up to a value of 13.0 ± 1.4 x 10^—6^ a.u. (n = 3), which clearly pointed to the formation of a highly viscoelastic layer most probably comprising subtilisin A molecules on the S-layer lattice. At the time of 250 min, both Δ*f* and Δ*D* increased indicating that subtilisin A started to cleave the SLP, and hence, protein is removed from the sensor surface (indicated by Δ*f*) and the remaining film became more viscoelastic (indicated by Δ*D*). At time point 270 min, rinsing with buffer A caused the spikes in both traces and a pronounced detachment of SbpA (Fig. 2). Finally, all the SLP was removed from the surface as both the frequency and dissipation increase and decrease, respectively, to their initial values observed with the plain sensor surface. These data clearly show the proteolytic action of subtilisin A as it was able to hydrolyze and cleave off the surface-attached SLP from the sensor.

### B Lipid-covered S-layer lattice

**Fig. 3:**
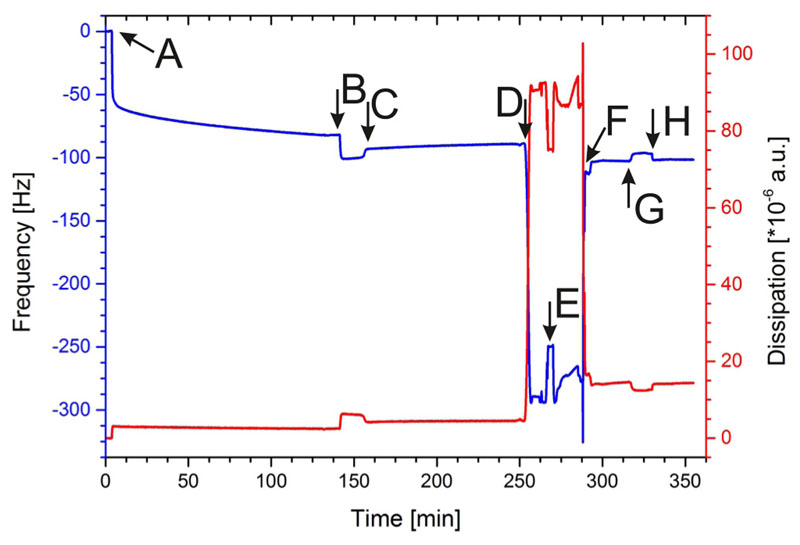
Shift in frequency (blue) and dissipation (red) during the formation of the S-layer supported lipid membrane and the subsequent treatment with subtilisin A. (A) Injection of the SbpA solution; (B) activation of SbpA; (C) addition of anchor lipids (DMPE); (D) rinsing with 70% ethanol; (E) addition of membraneforming lipids (DOPC) in ethanol; (F) rapid solvent exchange from organic to aqueous phase to form a membrane; (G) addition of subtilisin A; and (H) rinsing with HEPES buffer.

Pumping SbpA into the QCM-D measuring cell led to an immediate shift in frequency and dissipation (Fig. 3, point A). The recrystallization process of the SLP was finished after approximately 90 min. At point B (Fig. 3), the carboxyl groups of the SLP were activated by the addition of EDC/S-NHS to allow the covalent binding of the terminal amine groups of the DMPE molecules. After DMPE was injected, the frequency slightly increased (Fig. 3, point C), which is due to the change in buffer conditions. Figure 1(c) shows a schematic drawing of the sparsely bound DMPE molecules to the SbpA layer.

Subsequently, this structure was rinsed with 70% ethanol (Fig. 3, point D), which resulted in a very pronounced decrease in Δ*f* and increase in Δ*D*, respectively. This does not indicate a mass adsorption or change in viscoelasticity of the attached layer but reports on the change from an aqueous to an organic solvent. At point E (Fig. 3), DOPC dissolved in 70% ethanol was passed over the sensor surface. After exchange of the ethanol to an aqueous condition by flushing HEPES over the sensor surface, Δ*f* increased and AD decreased again (Fig. 3, point F). One can conclude the formation of a lipid membrane from the difference in frequency of the SbpA layer with (Fig. 3, point F) and without (Fig. 3, point B) the membrane. The calculation of Δ*f* resulted in a value of —20 ± -4 Hz (n = 3), which is in accordance with data from the literature.^48–50^ Moreover, AD of 12 x 10^—6^ ±2x10^—6^ a.u. is in accordance with previously published data for an S-layer supported lipid membrane generated by the RSE method.^41^ Although this AD value is higher compared to membranes on silicon dioxide,^48,49^ one has to keep in mind that the adjacent lipid leaflet is immobilized only by few lipid head groups to the SLP lattice. Hence, the intrinsic feature of this structure (i.e., sparsely anchored adjacent lipid leaflet with a floating second leaflet on the top) gives rise to a semifluid, viscoelastic membrane.^6,51,52^

At point G (Fig. 3), subtilisin A in buffer A was injected, and after rinsing with HEPES buffer (point H), no change in Δ*f* and Δ*D* with respect to the values before subtilisin A addition was observed. This result provides evidence that the lipid membrane totally shields the underlying S-layer lattice, and hence, subtilisin A cannot hydrolyze the underlying SbpA. To conclude, a closed lipid membrane without holes, which would allow subtilisin A to attack the SLP, has successfully been formed on the S-layer lattice by the RSE method.

## Hybrid polymer/lipid-covered S-layer lattice

In this set of experiments, the phospholipid DOPC was mixed with the polymer PBD-PEO in a molar ratio of 7:3. A lower molar lipid content was not chosen because FRAP measurements revealed a drastically reduced probe diffusion constant for hybrid membranes with a higher block copolymer content of 30%.^34^ The further procedure was the same as described previously (Sec. III B).

**Fig. 4:**
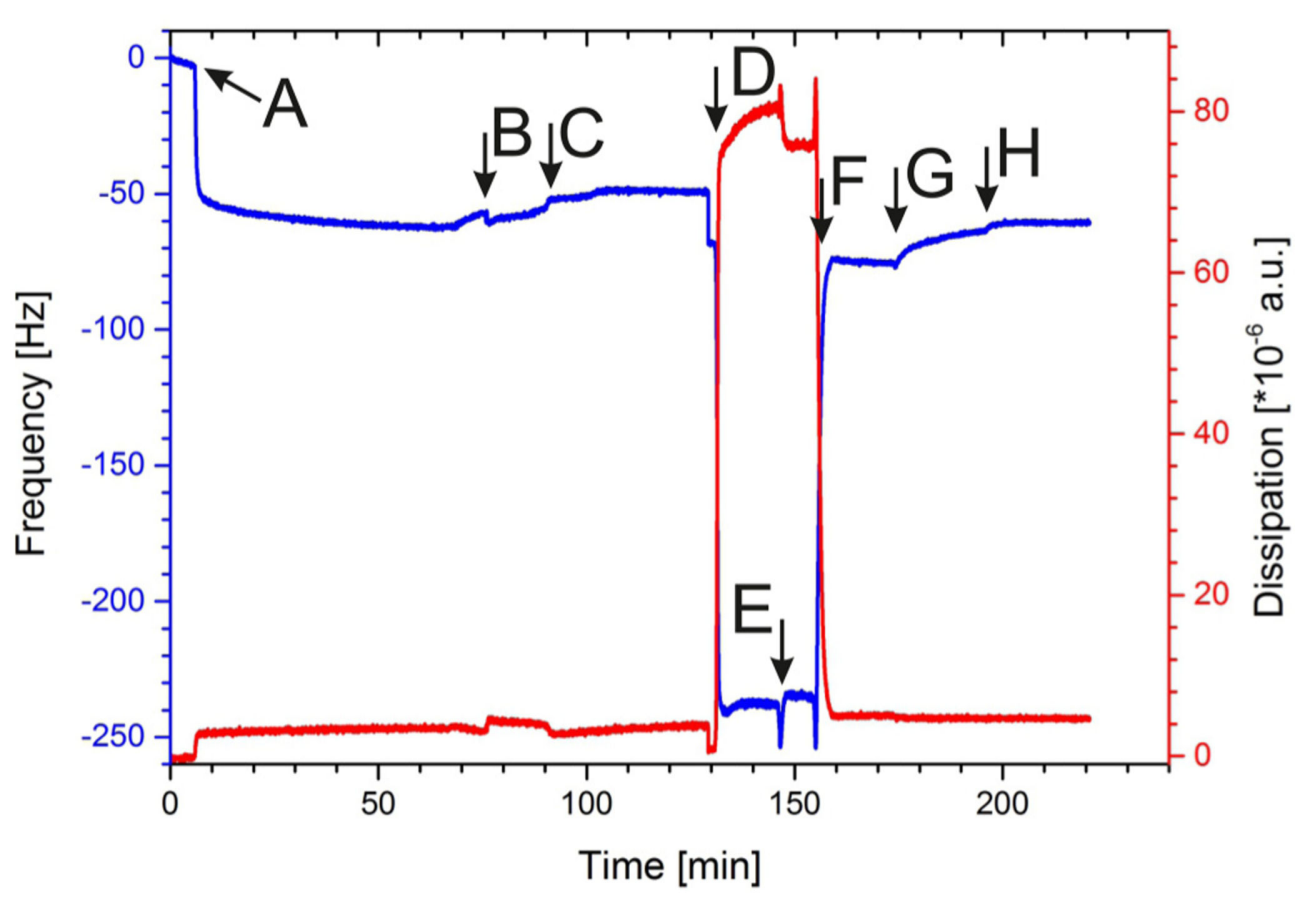
Shift in frequency (blue) and dissipation (red) during the formation process of the S-layer supported mixed lipid/polymer membrane and the subsequent treatment with subtilisin A. (A) Injection of the SbpA solution; (B) activation of SbpA; (C) addition of anchor lipids (DMPE); (D) rinsing with 70% ethanol; (E) addition of lipid/polymer mixture (DOPC/PBD-PEO) in ethanol; (F) rapid solvent exchange from organic to aqueous phase to form a hybrid membrane; (G) addition of subtilisin A; and (H) rinsing with HEPES buffer.

As shown in Fig. 4, SbpA was injected at point A and the recrystallization was finished after approximately 90 min. At point B (Fig. 4), the carboxyl groups of the SLP were activated by EDC/ S-NHS to allow the binding of the terminal amino groups of the DMPE molecules. After DMPE was injected, the frequency slightly increased (Fig. 4, point C), which is due to buffer exchange.

Subsequently, this structure was rinsed with 70% ethanol (Fig. 4, point D), which resulted in a very pronounced decrease in Δ*f* and increase in Δ*D*, respectively. Again, these shifts report on the change from an aqueous to an organic solvent. Subsequently, the DOPC/PBD-PEO mixture dissolved in ethanol was passed over the sensor surface (Fig. 4, point E). After changing from the ethanolic condition back to an aqueous one by flushing with HEPES, Δ*f* increased and Δ*D* decreased again (Fig. 4, point F). The formation of a hybrid polymer/lipid membrane can be concluded from the difference in frequency of the SbpA layer before and after the RSE procedure, which is again in the range of —20 ± —4 Hz (n = 3). The shift in Δ*D* of 2 x 10^—6^ a.u., however, is a strong indication that the hybrid polymer/lipid membrane on the S-layer lattice [Fig. 1(d)] possessed a significant lower viscoelasticity compared to the pure lipid membrane with a AD of 12 x 10^—6^ ±2x10^—6^ a.u. Hence, the PBD-PEO polymer makes the hybrid membrane stiffer. The schematic drawing in Fig. 1(d) indicated a membrane with separated domains of lipid and polymer. This structural feature has been observed for vesicles composed of lipid/polymer mixtures.^53^ However, the structure of the presently formed planar hybrid polymer/lipid membrane has not yet been determined as this was not possible by QCM-D measurements. Further investigation, e.g., by microscopical and electrochemical techniques has to be performed to elucidate this issue.

At point G (Fig. 4), the subtilisin A solution was injected, and after rinsing (Fig. 4, point H), only a slight increase in Δ*f* was observed, whereas Δ*D* remained constant. Most importantly, the value for frequency did by far not decrease to a lower negative Δ*f* value, which would indicate the removal of the SLP from the sensor surface. Indeed, this result provides evidence that the hybrid polymer/lipid membrane shields the S-layer lattice from subtilisin A attack, and hence, the latter cannot hydrolyze the underneath SLP lattice recrystallized on the sensor surface. To sum up, by means of the RSE method, the generation of a closed hybrid lipid/ polymer membrane without holes on the S-layer lattice is feasible [see Fig. 1(d)].

## IV Summary And Conclusions

The present study provides evidence for the successful formation of a mixed, hybrid lipid/polymer membrane with a high polymer content of 30% on an S-layer lattice. This study opens up the opportunity to use surface-based techniques to investigate block copolymer membranes and their hybrids as well as to integrate them in surface-based applications. The formed lipid/polymer membrane showed the same shift in Δ*f* compared to the pure lipid membrane, indicating that both membranes have approximately the same average mass per area. This is conceivable because DOPC has a lower molecular weight but also a lower area per molecule than the polymer. Thus, less polymer molecules with a higher molecular weight are necessary to build up the same membrane area. The value for Δ*D*, however, is significantly higher for the pure lipid membrane compared to the hybrid lipid/polymer membrane both anchored to an S-layer lattice. As the dissipation is a measure for the viscoelastic properties of a surface-bound layer, this result clearly shows a higher stiffness of the hybrid lipid/polymer membrane. Most interestingly, both the pure and hybrid membrane revealed a barrier function against the proteolytic action of subtilisin A to the S-layer protein, which, in turn, proves the formation of hole-free membranes on the underlying S-layer lattice. This study describes, to our knowledge, the first successful for-mation of a planar hybrid polymer/lipid membrane generated by the RSE technique on a solid support, in particular, on a proteinaceous S-layer lattice.

The capabilities of supported lipid membranes have already opened the door to biotechnology applications in medicine, diagnostics, sensor systems, environmental monitoring, and energy storage.^5,54–58^ Integration of the planar S-layer supported hybrid lipid/polymer membrane with nonliving systems, e.g., biosensors, field effect transistors, or (micro) total analysis systems, constitutes promising bioinspired systems to bridge the worlds of conventional engineering and biology, and could strongly contribute to the development of both. Thus, it may be possible to fabricate novel optic, electronic, high-throughput screening, drug targeting and delivery, sequencing, and sensory micro- and nanosystems by enhancing current bottom-up techniques.^1,5^
